# Porcine Cysticercosis and Risk Factors in The Gambia and Senegal

**DOI:** 10.1155/2010/823892

**Published:** 2010-08-12

**Authors:** Arss Secka, Tanguy Marcotty, Redgi De Deken, Eric Van Marck, Stanny Geerts

**Affiliations:** ^1^International Trypanotolerance Centre, PMB. 14, Banjul, Gambia; ^2^Department of Animal Health, Institute of Tropical Medicine, Nationalestraat 155, 2000 Antwerp, Belgium; ^3^Department of Veterinary Tropical Diseases, Faculty of Veterinary Sciences, University of Pretoria, Private Bag X04, Pretoria 0002, South Africa; ^4^Laboratory of Pathology, Faculty of Medicine, University of Antwerp, 2000 Antwerp, Belgium

## Abstract

During a stratified cross-sectional survey, 1705 pigs were sampled from 279 randomly selected households, 63 randomly selected communities and villages, from four study areas in The Gambia and Senegal during the period October 2007 to January 2008. Porcine cysticercosis prevalence detected by tongue inspection at animal level per study area ranged from 0.1% to 1.0%. Using an antigen-detection ELISA the seroprevalence of cysticercosis at both community/village and animal levels for the four selected study areas is: Western region 80.0% (95%CI: 52.4%–93.6%) and 4.8% (95%CI: 3.4%–6.5%), Bignona 86.7% (95%CI: 59.8%–96.6%) and 8.9% (95%CI: 5.0%–15.5%), Kolda 82.4% (95%CI: 46.8%–96.1%) and 13.2% (95%CI: 10.8%–16.0%), and Ziguinchor 81.3% (95%CI: 43.5%–96.1%) and 6.4% (95%CI: 4.0%–10.1%), respectively. No risk factors for cysticercosis were found significant in this study. This study proved that porcine cysticercosis is endemic and distributed widely in the study areas though its incidence might be suppressed by the generalised use of toilets and latrines in the study areas.

## 1. Introduction

Porcine cysticercosis caused by the larval form (cysticercus or metacestode) of *Taenia solium* is a parasitic disease of both economic and public health importance. Although rarely associated with clinical symptoms [1], porcine cysticercosis causes great economic losses due to the disposal or the processing of infected carcasses. In Mexico, the disease causes a loss of more than half the national investment in swine production and an annual economic loss of US$ 164 million in Latin America [2]. In the same country, it was also reported that cysticercosis caused pig production losses of US $43 million in 1980 [3]. Annual losses due to porcine cysticercosis in 10 West and Central African countries are estimated at 25 million Euros [4]. All these figures are gross estimates, and more research is necessary to calculate the real economic cost of porcine cysticercosis. The public health importance of *T. solium* is linked to the fact that humans are the definitive hosts of the parasite, but more importantly they may develop neurocysticercosis by the accidental ingestion of the tapeworm eggs. Neurocysticercosis, which is one of the major causes of epilepsy in developing countries [1], is a neglected disease, and its prevalence is largely underestimated [5].

There is limited information on the prevalence of porcine cysticercosis in The Gambia and Senegal. It has not been reported to the World Organisation for Animal Health (OIE) by both countries from 1996 to 2009 [6], and published articles on porcine cysticercosis from these countries are scanty. One report on data from six abattoirs in the Cap-Vert region of Senegal about causes of pig carcases condemnation during 1971–1980 showed that 0.02% had cysticercosis [7]. This information, however, is quite old and does not estimate the prevalence and risk factors of the disease in live pigs. Therefore, this survey was implemented to estimate the prevalence and assess the risk factors of porcine cysticercosis in selected areas in The Gambia and Senegal. 

## 2. Materials and Methods

### 2.1. Study Areas, Sampling Procedure, and Sample Size

**Figure 1:**
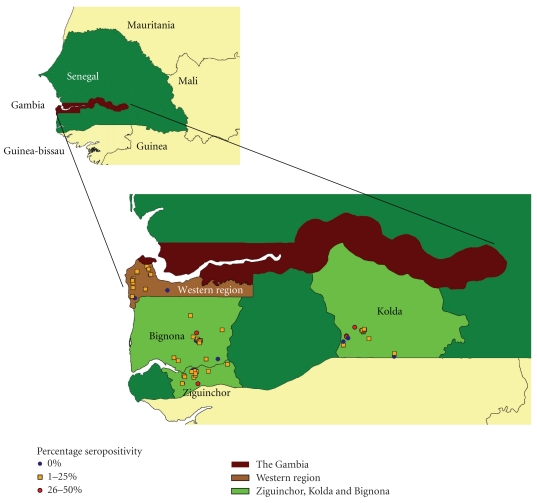
Porcine cysticercosis seroprevalence at community and village levels in the four study areas in The Gambia and Senegal.

Four study areas comprising of communities, villages, and towns within a 30 km radius per area were chosen for this cross-sectional survey based on their adjacency and higher pig population than other areas. These are Western region (WR) of The Gambia and Bignona, Kolda, and Ziguinchor departments in southern Senegal (Figure 1). Pig production in The Gambia and Senegal is localised in non-Muslim communities that form only 10% of the population. The majority of the pigs are raised under a free-range husbandry system, and only few are under semi-intensive or intensive system. Estimates of the pig population in Gambia and Senegal have shown a steady increase from 16,000 and 308,549 in 2005 to 25,000 and 325,747 heads in 2008, respectively [8].

A pig census was carried out in the households of the selected study areas during July-August 2007 to produce a sampling frame that would facilitate the sampling process. Field sampling for serum sample and data collection was implemented in October 2007–January 2008. The sampled communities, villages, and households were selected at random using computer generated numbers in MS Excel. 

In Western region (WR) of The Gambia, the villages (primary sampling units) were grouped into three strata according to their type of pig management system. The first group consisted of villages that permanently confine pigs, group two were villages that seasonally confine pigs (only during the rainy season), and group three did consist of both types of confinement. Five villages were randomly selected from each group, two to six households were randomly selected per village, 21 to 25 pigs were sampled per village, and finally 371 pig samples were collected (Table 1). 

In Southern Senegal, pigs are permanently or seasonally confined across all villages and communities in the three study areas. All villages in each study area were stratified as rural, and all the communities in each commune (administrative départemental capital) were stratified as urban. At rural strata level, villages were first selected at random, then households were selected randomly, and finally sampled pigs selected. Similarly, at urban strata (commune) level: communities (quartiers) were first selected at random, then households were selected at random, and finally sampled were pigs selected (Table 1). 

Selection of household pigs was not at random. Only accessible pigs more than 3 months old, nonlactating sows, and sows that were not in late gestation were sampled. Out of 22,821 pigs in the four study areas 1,705 pigs were sampled consisting of 1,050 females and 655 males, with 954 animals less than or equal 12 months and 751 more than 12 months old. The proportions of sampled pigs from the populations of pigs under seasonal confinement and permanent confinement were 8.3% and 7.2%, respectively. Ninety percent (1532) of the sampled pigs belonged to the local breed whilst the others were crossbreds of local with exotic breeds. 

Table 1 summarizes the pig population (July-August 2007) and sample size of the four study areas. The required sample sizes for this survey were calculated in Intercooled Stata 10 using a cysticercosis seroprevalence (Ag-ELISA) of 10% for Western region, and 12% for the areas in southern Senegal obtained from a preliminary survey (December 2006–March 2007) of slaughter pigs at Western region and Ziguinchor, respectively. The sample sizes to discriminate proportions of 10 and 12% from 5 and 7% (corresponding to an absolute estimate precision of 5%) were 301 and 372, respectively (with two-sided *α* = 5% and power = 90%). These figures ignored intracluster correlations and were increased to *n* (total pigs sampled) in Table 1 to get a minimum of 25 sampled pigs per village from Western region and 30 sampled pigs per village/community as far as possible.

### 2.2. Sample Collection and Storage

 About 5 ml of blood were collected from the jugular vein of large pigs or anterior vena cava of small pigs into plain tubes with clot activator. After centrifugation the serum from each tube was transferred into two labelled cryovial tube aliquots for each pig and stored at −20°C until tested.

### 2.3. Diagnostic Tests

Two cysticercosis diagnostic tests were applied during this survey. The first one was tongue inspection. The sampled pigs were restrained in a standing position by placing a wire snare behind the incisors of the upper jaw, the tongue pulled out, and examined visually for cysts. Pigs with cyst(s) on their tongue were considered infected with cysticercosis. The second test was a monoclonal antibody-based sandwich enzyme-linked immunosorbent assay (Ag-ELISA) to detect circulating antigens of *Taenia solium* [9, 10]. The serum samples were first treated with trichloroacetic acid (TCA) to break the antigen-antibody complexes and then tested at a final dilution of 1 : 4. Briefly the sandwich assay consisted of coating the plates with capturing antibody (B158C11A10), blocking, addition of TCA treated sera, after which the second biotin labelled antibody (B60H8A4), streptavidin labelled peroxidase and ortho phenylenediamine (OPD) substrate were added consecutively. Washings were carried out in between the various steps. The reaction was stopped using sulphuric acid and the plates were read in a spectrophotometer at a wavelength of 492 nm. The cutoff was calculated using a modified Student *t*-test [11] programmed in MS Excel sheet, by comparing the optical density of each serum sample with a series of 8 negative serum samples obtained from a commercial pig farm without any history of cysticercosis in The Gambia at a probability level of *P* < .001. A serum sample was considered as positive when the ratio (optical density of test sample/optical density cut-off) was ≥1.0. 

### 2.4. Data Collection and Analysis

Questionnaires were administered by the first author (Arss Secka) using a combination of English, French, and “Wollof”, a local language. The household heads and/or members served as respondents during a face-to-face interview. The administered questionnaire was structured to gather data about the characteristics of the household, pig management, sanitation and hygiene, knowledge on cysticercosis transmission and occurrence, epileptic seizures, pig sales, and occurrence of *T. hydatigena* (large fluid-filled vesicles in the peritoneal cavity). For every sampled pig, information about the pig's confinement method, access to human faeces, sex, age, household identity, village/community, strata, study area, and availability of a household toilet was also recorded.

All statistical analyses were done separately for Gambian and Senegalese data using logistic regressions in Stata 10. Binary Ag-ELISA data resulting from stratified sampling were first analysed in a stepwise backward selection of estimators process (*P* < .1) to discard insignificant explanatory variables. The discrete explanatory variables included “département” or region, pig's sex, age class, access to human faeces, management, availability of household toilet, and urbanisation. The retained explanatory variables were subsequently tested in robust survey models accounting for strata (urban/rural/group), primary (village and community), and secondary (household) sampling units, finite population correction, and sampling weights. The department seroprevalences and confidence intervals were calculated using the same robust models but “department” was the only explanatory variable. Village seroprevalences were calculated in similar robust models but only accounting for strata. A village was considered infected when one pig at least was positive in the Ag-ELISA test. Finally, tongue infection data from individual pigs were also analysed in robust logistic regressions using the same strata, primary, and secondary sampling units, finite population correction, and weights as above.

## 3. Results

### 3.1. Household Questionnaire Results

Across the four study areas, 279 households with pigs and 63 communities and villages were involved in the study. All households were Christian except one Muslim household in Ziguinchor. The Manjago ethnic group was predominant in Western region households (69.6%), Balanta in Kolda (41.2%), and Jola in Ziguinchor and Bignona (62.4% and 87.8%, resp.). Fifty five percent of these households were involved in farming. 

All households except two in Western region consumed pork in barbecued or cooked form. Although 64.5% and 6.5% of the households knew porcine and human cysticercosis, respectively, none of the household representatives knew how it is transmitted. Cysticerci in slaughter pigs were observed during the period 1987–2003 by 11.5% of the households. Although questionable, respondents reported that infected carcases were thrown away. Persons reporting to have manifested epileptic seizures were present in 7.2% of the households. Almost all households had toilets that were used regularly except for 5% that used the bush and 2% that used neighbour's toilet. Whereas 76% of the households practised seasonal confinement, pigs belonging to 48.8% of the households had access to human faeces. Within the year 2007, 129 households reported to have sold 635 pigs of which 148 (23.3%) were sold to traders from the Republic of Guinea Bissau and the rest to local consumers in the study areas. Occurrence of *T. hydatigena *cysts in slaughtered pigs was reported neither by butchers nor by farmers and was not observed in Western region and Ziguinchor during the preliminary porcine cysticercosis survey and this cross-sectional survey.

### 3.2. Cysticercosis Seroprevalence and Risk Factors

One to two pigs were found positive by tongue inspection in each of the study areas, whilst the highest and lowest cysticercosis seroprevalences at animal level were found in Kolda “départment” and Western region, respectively (Table 2).

Analyses of the data from Western region showed that none of the explanatory variables (group, pig sex, age, management, and access to faeces) were retained by the stepwise backward selection of estimators. However, analyses of data from southern Senegal gave different results. The stepwise backward selection retained “départment”, pig management, and access to faeces (*P* ≤ .001,  .007, and.036, resp.). Pig's sex and age class, urbanisation, and availability of household toilet were not significantly associated to the occurrence of cysticercosis (positive Ag-ELISA test). After testing the retained variables (“départment”, pig management, and access to faeces) in a robust survey model, only Kolda département had a significantly higher cysticercosis occurrence (positive at Ag-ELISA test) than Bignoma (OR = 0.44; 95% CI: 0.23–0.84) and Ziguinchor (OR = 0.30; 95% CI 0.13–0.68). The occurrence of cysticercosis in Senegal was not significantly lower when pigs were seasonally confined (OR = 0.66; 95% CI: 0.33–1.31) (pigs confined in July–October) nor was it significantly higher when pigs had access to human faeces (OR = 1.5; 95% CI: 0.83–2.8). Figure 1 shows the map of the four study areas and percentage of animal seropositivity to cysticercosis at sampled communities and village. 

## 4. Discussion

### 4.1. Porcine Cysticercosis Prevalence

This is the first report of a porcine cysticercosis study that utilized a cross-sectional survey approach, serology, and tongue inspection in live pigs in both The Gambia and Senegal. Tongue inspection detected one to two cases of porcine cysticercosis in each study area, which tends to indicate that there might be few pigs with massive *T. solium* infections in the region. It is well known that tongue inspection is likely to detect just the mostly heavily infected pigs and that it is an effective and quick screening method in hyperendemic areas [12]. However, whereas it is reported to have a high specificity (100%), this diagnostic method lacks sensitivity (21%) [10]. The values of cysticercosis prevalence found in this study using tongue inspection are lower than values reported in other studies: 5.2 to 10.9% in Zambia [13], 11.9% in Eastern Cape Province, South Africa [14], 4.4% in North-West Cameroon [15], and 17.4% in Mbulu district Tanzania [16]. It appears that the cysticercosis infections in pigs in this study are rather light as compared to other African countries. 

Cysticercosis seroprevalence at community and village level however, is very high. This shows how widespread the infection is among the sampled communities and villages. However, the seroprevalence at animal level is not as high as at community/village level, and also lower than in other reports. A seroprevalence of porcine cysticercosis ranging from 11.0 to 39.8% using Ag-ELISA was found in four areas in Cameroon [4], 27.7% in North-West Cameroon [15], and 64.2% using four tests in a Bayesian approach in Zambia [10]. The lower values found in this study could be attributed mainly to lower infection rates in sampled pigs, but it is in agreement with reports of below 10%–20 % prevalence rate for West Africa [17].

The Ag-ELISA, which has been validated by postmortem examination [10, 18], has a sensitivity of 86.7% and specificity of 94.7%. It is considered as the most sensitive test to detect cysticercosis in pigs, but it cross-reacts with *T. hydatigena* [10]. The monoclonal antibodies used in this Ag-ELISA were prepared against the excretory-secretory antigens of *T. saginata* [9] hence it is rather genus than species specific. The seroprevalence figure of this study, however, can be considered reliable since *T. hydatigena* seems to occur only sporadically in pigs in the region. 

### 4.2. Porcine Cysticercosis Risk Factors

This study failed to confirm that seasonal confinement and access to human faeces are important risk factors for porcine cysticercosis. This is in contradiction with the observations of Shey-Njila et al. [15] in North-West Cameroon who reported a higher seroprevalence of porcine cysticercosis in households without latrines and in households who have pigs which are not permanently confined. Free ranging of pigs, lack of latrines, home slaughtering of pigs, absence of pork inspection, and barbecuing were found as risk factors for porcine cysticercosis in the southern highlands of Tanzania [19]. Free-range pig husbandry is also a significant risk factor for porcine cysticercosis in Eastern and Southern provinces of Zambia [20]. Probably due to the presence of toilets in 93% of sampled households and absence of human defecation in pig pens in our study areas, most of the risk factors reported for North-West Cameroon and the southern highlands of Tanzania were not found in this study. 

Although 93% of the surveyed households have toilet facilities, the fact that 76% of the sampled pigs are only seasonally confined means that these pigs might come into contact with taeniid eggs in the environment. The five percent of sampled households that report to use the bush for defecation might be responsible for the environmental contamination assuming that there are tapeworm carriers among them. The risk for pigs to come into contact with taeniid eggs, however, is reduced by the high number of households with toilet facilities in usage. This might explain why the seroprevalence of cysticercosis found in this study is rather low as compared to findings in other African countries. 

There are no pig abattoirs, and there is no inspection of porcine carcases by the veterinary or medical personnel at the four study areas except for a limited coverage in Ziguinchor. Many pigs are slaughtered for consumption during family festivities or sold to customers on demand without any carcase inspection. Under this kind of situation where evidence of porcine cysticercasis has been demonstrated, humans are at risk of getting taeniosis/cysticercosis. Soutou is one of the sampled villages in the Bignona département in Senegal where a seroprevalence of 26.7% porcine cysticercosis was found. There are reports of an outbreak of *T. solium* cysticercosis in this village with 23 human cysticercosis cases in the 1960s [21] and two extra cases in the seventies [22]. Half a century later, this village might still be harbouring human cysticercosis cases as a high porcine cysticercosis prevalence is indicative of environmental contamination with taeniid eggs. A study is currently going on to examine this further in detail.

Cross-border trade in live animals has the risk of bringing transmissible diseases into the recipient countries. Trade in pigs from the study areas to the Republic of Guinea Bissau seems important since 23.3% of pigs sold in 2007 by the sampled households in Senegal were destined to the Republic of Guinea Bissau. Although the cysticercosis status of the sold pigs was unknown, it is probable that infected pigs might have been exported to Guinea Bissau. 

In conclusion this study has demonstrated the endemic occurrence of porcine cysticercosis in four areas of The Gambia and Senegal. Kolda département, with higher number of households using the bush for defecation, showed significantly higher infection rates than the “départements” of Bignona and Ziguinchor and Western region. Although the prevalence of porcine cysticercosis appears to be lower than in other African countries, there should be no complacency on control efforts. It is well known that in areas where porcine cysticercosis is present people are at risk of taeniasis and neurocysticercosis. Both veterinary and medical public health services should determine foci of the disease transmission and devise control strategies to reduce the disease risk in both Senegal and The Gambia. 

## Figures and Tables

**Table 1:** Pig population (*N*) and sample size (*n*) of the four study areas in The Gambia and Senegal.

Region/Department	Total villages/communities		Total households		Total pigs		Pig confinement type			
							Seasonal		Permanent	
	*N*	*n*	*N*	*n*	*N*	*n*	*N*	*n*	*N*	*n*
Western region	**44**	**15**	**565**	**69**	**9234**	**371**	**3223**	**185**	**5223**	**186**
Group one	12	5	165	23	3116	125	0	0	3116	125
Group two	11	5	122	23	1495	121	1495	121	0	0
Group three	21	5	278	23	4623	125	2043	64	2580	61
Bignona	**28**	**15**	**417**	**74**	**4044**	**433**	**3888**	**318**	**139**	**115**
Rural	24	10	327	51	3445	300	3369	236	79	65
Urban	5	5	90	23	1599	133	519	82	80	50
Kolda	**18**	**17**	**78**	**51**	**2728**	**449**	**2593**	**417**	**135**	**32**
Rural	9	9	14	14	219	116	210	115	9	5
Urban	9	8	87	37	2509	333	2383	302	126	27
Ziguinchor	**34**	**16**	**754**	**85**	**6823**	**452**	**6246**	**353**	**577**	**99**
Rural	20	8	541	43	4368	218	4347	199	21	19
Urban	14	8	213	42	2455	234	1899	154	556	80

Total	**125**	**63**	**1797**	**279**	**22821**	**1705**	**15191**	**1266**	**6072**	**439**

**Table 2:** Porcine cysticercosis prevalence by tongue inspection and seroprevalence by Ag-ELISA in four study areas in The Gambia and Senegal.

Region/“département” ^†^	Tongue inspection	Ag-ELISA test	
	Prevalence at animal level	Seroprevalence at community/village level *	Seroprevalence at animal level
Western	0.2% (CI: 0.0–1.1)	80.0% (CI: 52.4–93.6)	4.8% (CI: 3.4–6.5)
Bignona	1.0% (CI: 0.2–4.1)	86.7% (CI: 59.8–96.6)	8.9% (CI: 5.0–15.5)
Kolda	0.1% (CI: 0.0–0.3)	82.4% (CI: 46.8–96.1)	13.2% (CI: 10.8–16.0)
Ziguinchor	0.3% (CI: 0.0–1.4)	81.3% (CI: 43.5–96.1)	6.4% (CI: 4.0–10.1)

CI: 95% confidence interval; ^†^“département” is the second largest local administrative unit in Senegal comprising of a group of towns and villages; *seroprevalence at community/village level is the proportion of sampled communities and villages in which at least one pig was found positive in the Ag-ELISA.
